# Adherence to Mediterranean diet associated with health-related quality of life in children and adolescents: a systematic review

**DOI:** 10.1186/s40795-022-00549-0

**Published:** 2022-06-23

**Authors:** Milton A. Romero-Robles, Fabricio Ccami-Bernal, Zhamanda N. Ortiz-Benique, Diego F. Pinto-Ruiz, Vicente A. Benites-Zapata, Donovan Casas Patiño

**Affiliations:** 1 Escuela de Medicina, Universidad Nacional del Santa, Nuevo Chimbote, Ancash, Perú; 2 Facultad de Medicina, Universidad Nacional de San Agustín de Arequipa, Arequipa, Perú; 3 Facultad de Medicina, Universidad Nacional Mayor de San Marcos. Lima, Lima, Perú; 4 Doctorado en Nutrición Y Alimentos, Universidad San Ignacio de Loyola. Lima, Lima, Perú; 5 Universidad Autónoma del Estado de México. Red Internacional en Salud Colectiva Y Salud Intercultural, Toluca, México

**Keywords:** Diet, Mediterranean, Quality of life, Child, Adolescent, Patient Compliance (MeSH—NLM)

## Abstract

**Background:**

Health-related quality of life (HRQoL) has become a significant outcome in assessing interventions in the pediatric population and could be influenced by diet patterns. The Mediterranean diet (MD) pattern has been related to multiple positive health outcomes, including decreased cardiovascular risk and better mental health. We aimed to evaluate the association between MD adherence and HRQoL in children and adolescents.

**Methods:**

The literature search was conducted in PubMed, Cochrane Library, Scopus, Web of Science, Embase, and Ovid-MEDLINE databases from inception to May 2022. Two researchers independently checked titles and abstracts, evaluated full-text studies, extracted data, and appraised the risk of bias using the Newcastle–Ottawa Scale (NOS).

**Results:**

Eleven studies (1 longitudinal and 10 cross-sectional), totaling 6,796 subjects, were included. Ten studies assessed MD adherence with KIDMED index, and one assessed MD adherence with Krece Plus test, while all included studies assessed HRQoL with a KIDSCREEN test. All studies analyzed the association between MD adherence and HRQoL with linear regression, and eight used adjusted models. Five studies found a significant positive association of MD adherence with HRQoL, with β-values ranging from 0.13 to 0.26. Two found a nonsignificant positive relationship, while one found a negative association. According to the NOS criteria, the risk of bias assessment showed four studies with a low risk of bias and seven with a high risk of bias.

**Conclusion:**

Our findings suggest a positive correlation of MD adherence with HRQoL in children and adolescents. However, future research is needed to strengthen the evidence of this relationship.

**Trial registration:**

CRD42021236188 (PROSPERO)

**Supplementary Information:**

The online version contains supplementary material available at 10.1186/s40795-022-00549-0.

## Background

The World Health Organization (WHO) defines Quality of Life (QoL) as an individual's perception of position in life in the context of the culture and value systems in which they live and concerning goals, expectations, standards, and concerns [1]. To differentiate terminology in this paper, we regard QoL in children as a multidimensional subjective concept that includes social, emotional, cognitive, and physical functioning as well as cultural aspects of the child and family. At the same time, Health-Related Quality of Life (HRQoL) incorporates measures of physical symptoms, functional status, and disease impact on psychological and social functioning. This endpoint has been extensively studied in recent years to assess the efficacy of health services and interventions in the pediatric population, turning the attention from school attendance to this outcome [2, 3]. In this way, HRQoL assesses the children more integrally, including physical symptoms, psychological health, and socioeconomic environment [3].

One of the factors of QoL is diet, understood as the dietary regimen or eating habits—the composition, frequency, and amount of food and beverages consumed during the day. These are social practices where culture, traditions, customs, habits, policies, the norms, and values ​​of each social group construct and reconfigure "healthy" dietary patterns, such as the Mediterranean diet (MD), which is associated with better QoL scores on physical and mental dimensions [4]. There is no strict consensus on what constitutes a Mediterranean diet in percentages and macronutrients [5]. However, it is accepted that the Mediterranean dietary pattern includes moderate consumption of unsaturated fats, fish, lean meats, fruits, vegetables, nuts, legumes, and low consumption of red meat and saturated fats [6] Its consumption is associated with multiple beneficial health outcomes, such as preventing cardiovascular disease, reduced risk of certain types of cancer, and even better cognitive and mental health outcomes. However, the quality of the available studies is low to moderate [7]. These results come from studies conducted in the general population and mostly from European countries [8].

Although a systematic review has been carried out on the relationship between MD and QoL [2], so far, no systematic data have been produced on this topic in special populations such as those in the first two decades of life, where they are suffering from multiple health problems such as chronic non-communicable diseases -obesity, overweight, diabetes. In addition, the pediatric population tends to abandon the Mediterranean lifestyle, which, unfortunately, could lead to adverse health events [9] Therefore, knowledge of the benefits of the Mediterranean diet on QoL could be valuable for promoting adherence in these populations through health agencies and decision-makers. For this reason, the present study aimed to evaluate the association between adherence to the MD with HRQoL in children and adolescents.

## Methodology

### Protocol and registration

We performed a systematic review following the recommendations of the Cochrane Handbook for Systematic Reviews of Interventions [10] and reported according to the Preferred Reporting Items for Systematic Reviews and Meta-analyses (PRISMA 2020) recommendations [11]. The study protocol has been registered at PROSPERO (number CRD42021236188).

### Eligibility criteria

For this systematic review, we included randomized controlled trials (RCTs), quasi-experimental controlled, cohort, case–control, and cross-sectional studies that evaluated the association between adherence to the MD (autocompleted or completed with parent help) and HRQoL in children and adolescents (over 6 years old and under 18 years old), excluding population with comorbidities or some disease. In addition, we excluded the following type of studies: non-controlled studies, review articles, abstracts, case reports, letters, conference papers, or editorials.

### Information sources and search strategy

We searched the following databases: PubMed, Cochrane Library, Scopus, Web of Science, Embase, and Ovid-MEDLINE. The first search was conducted on February 10, 2021, and last updated on May 4, 2022. The search terms were adapted to each database. There were no restrictions on language or publication date. The complete search strategy for each database, number of studies included for full-text review, and reasons for exclusion were recorded and provided in Additional File 1. Moreover, we reviewed the references of included studies to find more potential eligible studies.

### Selection process

**Fig. 1:**
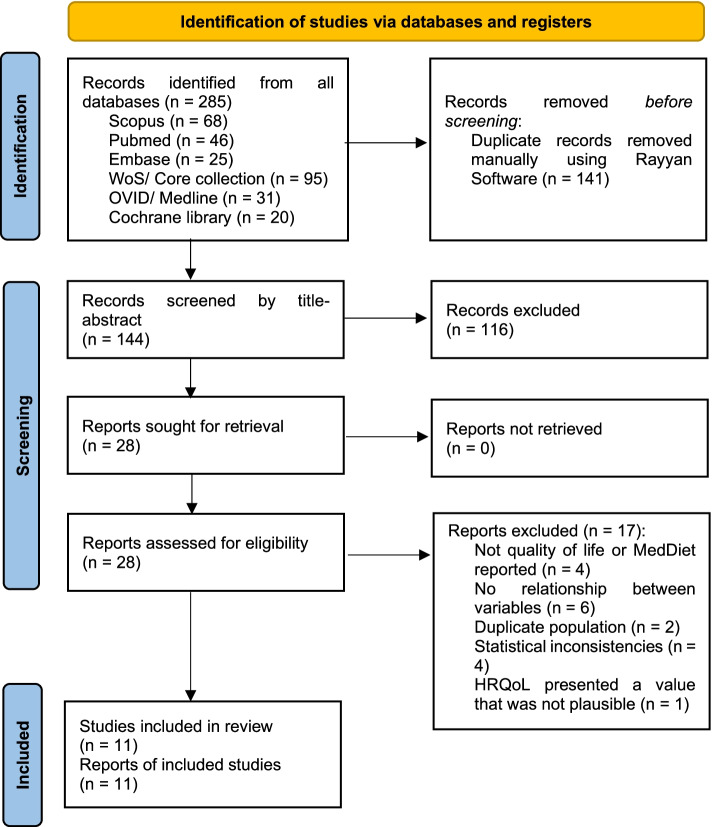
Flow diagram summarizing the process of literature search and selection

Duplicate articles were removed using the Rayyan web application, and then two reviewers (D.F.P.R. and Z.N.O.B.) screened titles and abstracts and identified potentially relevant studies for inclusion. These studies were read in full-text and evaluated for inclusion (M.A.R.R. and F.J.C.B.). These processes were performed independently, and a discussion resolved disagreements to achieve consensus. Any unresolved disagreements were evaluated by a third reviewer (V.A.B.Z.). This process will be recorded using a PRISMA flowchart, version 2020 (Fig. 1).

### Data collection process and data items

Four authors grouped in pairs (M.A.R.R. and F.J.C.B., D.F.P.R. and Z.N.O.B.) independently extracted the following information of the included studies into a Google Sheet: title, first author, year of publication, country, study design, context, inclusion criteria, sample size, participant characteristics (sex, age, weight, height, body mass index, abdominal circumference, physical activity, sedentary time, sleep time, obesity, and overweight), exposure (self-report-yes or no, instrument, dimensions, and score), outcome (self-report-yes or no, instrument, dimensions, and score), result, statistical method, covariates, and funding. A fifth reviewer evaluated any unresolved disagreements (V.A.B.Z.). In the case that an article did not present all its data, the corresponding author was contacted.

### Risk of bias

Four authors grouped in pairs (M.A.R.R. and F.J.C.B., D.F.P.R. and Z.N.O.B.) independently assessed the risk of bias. Because only observational studies were identified, we assessed the risk of bias with the adapted version of the Newcastle–Ottawa Scale (NOS) for cross-sectional studies [12]. The NOS assesses three domains: [1] selection (items: representativeness of the sample, sample size, nonrespondents, and ascertainment of the exposure), [2] comparability (item: comparability based on the study design or analysis), and [3] outcome (items: assessment of the outcome and statistical test). The NOS gives a maximum score of 10 points. We considered that a score of ≥ 7 meant a low risk of bias, a score between 4 and 6 meant a high risk of bias, and a score of < 4 meant a very high risk of bias [13]. Any unresolved disagreements were evaluated by a third reviewer (V.A.B.Z.).

### Statistical analyses

We did not perform the meta-analysis because of to the heterogeneity of the instruments used to assess adherence to the MD and HRQoL and insufficient data. It was also not possible to assess the risk of publication bias.

## Results

### Study selection

Our search strategy initially identified 288 articles. After removing 144 duplicates, 144 studies were screened in detail for eligibility. Our included ‘studies' eligibility process can be found in the flow diagram in Fig. 1. After screening titles and abstracts, 116 articles were excluded. After full-text revision of 28 articles, 17 articles were excluded because of the following: they included neither MD nor HRQoL (*n* = 4), the association between QoL and the MD was not investigated (*n* = 6), the publication of another study was already included (*n* = 2), there were statistical inconsistencies (*n* = 4) and there was one study in which HRQoL presented a value that was not plausible (see Additional File 2). Finally, 11 articles were included for qualitative synthesis [14–24].

### Studies and patients characteristics

Reports included were informed by data collected from 2012 to 2021. The studies were conducted in Spain [15, 18, 19, 21], Greece [14, 16], Chile [20, 22], Portugal [17], Lebanon [23] and Italy [24]. Ten studies had a cross-sectional design [14–17, 19–24], while one study was longitudinal [18]; therefore, it was considered the baseline cross-sectional cohort. The number of total participants included was 6,796 between 10 and 18 years old. The number of participants in the studies ranged from 114 to 1,523. The percentage of boys participants in the studies ranged from 44.5% to 55.9%, with females making up the remaining sample. Study characteristics are summarized in Table 1.

**Table 1:** Characteristics of the included studies

Author, publication year	Country	Study design	Total subjects	Inclusion criteria	Characteristics of participants				MD adherence scoring system ^‡^	MD adherence dimensions	HRQoL measure ^‡^	HRQoL dimensions	Adjusted variables	Funding
					**Gender and age**	**BMI (kg/m**^**2**^**)**	**Physical activity**	**Weight **^**†**^						
Costarelli et al., 2012 [14]	Greece	Cross-sectional	359 (13–16 years old)	Participants signed consent forms and received a full verbal and written explanation of the purpose of the study and its anonymous nature	46.2% boys Median age: 13.1 years (IQR: 0.5)	19.9 (SD, 3.2)	NR	Normal weight: 80.6% Overweight: 15.6% Obesity: 2.5%	KIDMED index	16 dichotomous items (yes/no): 12 items: behaviors consistent with the Mediterranean diet 4 items: behaviors inconsistent with the Mediterranean diet	KIDSCREEN-27	5 Dimensions: 1. Physical well-being (5 items) 2. Psychological well-being (7 items) 3. Parent relations and autonomy (7 items) 4. Social support and peers (4 items) 5. School environment (4 items)	Number of meals, number of meals (with family), BMI, obesity, father’s education level, mother’s education level, gender	Institutional support. Human Ecology Laboratory, Department of Home Economics and Ecology, Harokopio University of Athens
Muros et al., 2017 [15]	Spain	Cross-sectional	456 (11–14 years old)	Twenty-four adolescents were excluded for failing to complete some testing element or failing to attend class on their testing day	48.5% boys Mean age: 12.57 years (SD, 1.17)	19.75 (SD, 3.85)	PAQ-C score: 2.92 (SD, 0.64)	Normal weight: 75.7% Overweight: 16.7% Obesity: 5.5%	KIDMED index	16 dichotomous items (yes/no): 12 items: behaviors consistent with the Mediterranean diet 4 items: behaviors inconsistent with the Mediterranean diet	KIDSCREEN-27	5 Dimensions: 1. Physical well-being (5 items) 2. Psychological well-being (7 items) 3. Parent relations and autonomy (7 items) 4. Social support and peers (4 items) 5. School environment (4 items)	BMI, physical activity	NR
Zervaki et al., 2017 [16]	Greece	Cross-sectional	400 (14–17 years old)	Participants that signed consent forms received a full verbal and written explanation of the purpose of the study and its anonymous nature	49.5% boys Median age: 15.8 years (IQR: 1.03)	21.7 (SD, 3.1)	NR	Normal weight: 75.0 Overweight: 21.5 Obesity: 3.5%	KIDMED index	16 dichotomous items (yes/no): 12 items: behaviors consistent with the Mediterranean diet 4 items: behaviors inconsistent with the Mediterranean diet	KIDSCREEN-27	5 Dimensions: 1. Physical well-being (5 items) 2. Psychological well-being (7 items) 3. Parent relations and autonomy (7 items) 4. Social support and peers (4 items) 5. School environment (4 items)	Number of meals, number of meals (with family), age, obesity, father’s education level, mother’s education level, BMI	NR
Evaristo et al., 2018 [17]	Portugal	Cross-Sectional	946 (12–18 years old)	All students that agreed to participate in the study	53.3% boys Mean age: 14.5 years (SD, 1.8)	21.1 (SD, 3.8)	NR	NR	KIDMED index	16 dichotomous items (yes/no): 12 items: behaviors consistent with the Mediterranean diet 4 items: behaviors inconsistent with the Mediterranean diet	KIDSCREEN-10	It contains 10 items regarding family life, peers, and school life	Physical fitness, age, sex, pubertal stage (Tanners A and B), socioeconomic status, sleep duration, BMI	Portuguese Foundation for Science and Technology (FCT) grants
Esteban-Gonzalo et al., 2019* [18]	Spain	Cross-Sectional	(a) 536 children at primary school (6–7 and 9–10 years old) (b) 987 adolescents at secondary school (12–13 and 15–16 years old)	(i) To study in the first/fourth grades (6–7 and 9–10 years old, respectively) for children and seventh/tenth grades (12–13 and 15–16 years old, respectively) for adolescents at baseline and (ii) to not have physical disability or health problems, which may limit the levels of physical activity	Primary school: 49.8% boys Mean age: 8 years (SD, 1.5) Secondary school: 51.3% boys Mean age: 8.2 years (SD, 1.5)	NR	NR	Overweight and obesity Primary school***: ***Boys: 35.6% Girls: 36.9% Secondary school***:*** Boys: 39.0% Girls: 21.9%	KIDMED index	16 dichotomous items (yes/no): 12 items: behaviors consistent with the Mediterranean diet 4 items: behaviors inconsistent with the Mediterranean diet	KIDSCREEN-10	It contains 10 items regarding family life, peers, and school life	Age, socio-economic status, prevalence of overweight, and obesity	DEP 2010- 21,662-C04-00 grant from the National Plan for Research, Development and Innovation (R + D + i)
Ferrer-Cascales et al., 2019 [19]	Spain	Cross-Sectional	527 (12–17 years old)	NR	45.5% boys Mean age: 14.43 years (SD, 1.52)	NR	NR	NR	KIDMED index	16 dichotomous items (yes/no): 12 items: behaviors consistent with the Mediterranean diet 4 items: behaviors inconsistent with the Mediterranean diet	KIDSCREEN-52	10 Subscales: 1. Physical Well-Being 2. Psychological Well-Being 3. Mood and Emotions 4. Self-Perception 5. Autonomy 6. Parent Relations and Home Life 7. Financial Resources 8. Social Support and Peers 9. School Environment 10. Social Acceptance	NR	Self-funded
Delgado-Floody et al., 2020 [20]	Chile	Cross-Sectional	619 (10–13 years old)	(i) To be a student of a public school in the La Araucanía region, (ii) to have a regular physical education class and (iii) to be aged between 10 and 13 years. The exclusion criteria were as follows; (i) having musculoskeletal disorders or (ii) any other known medical condition, which may alter the participant’s health and PA levels; and (iii) to have scholars commitment that they could be interrupted by the study measurements	55.9% boys Mean age: 11.72 years (SD: 1.07)	21.57 (SD, 4.68)	NR	Normal weight: 50.4% Overweight: 25.2% Obesity: 24.4%	Krece Plus test	The questionnaire contains 15 items, where the maximum possible score was + 11 and the minimum was − 5. Each item has a score of + 1 or − 1, depending on whether it approximates the ideal of the MD. The total points are accounted and, according to the score, the nutritional status is classified as follows: (1) “low” nutritional level, ≤ 5; (2) “moderate” nutritional level, 6–8; and (3) “high” nutritional level, ≥ 9	KIDSCREEN-10	It contains 10 items regarding family life, peers, and school life	Age, sex	Research Project R01/18 from the Universidad de Los Lagos and by private fundings
Rodriguez-Rosado et al., 2020 [21]	Spain	Cross-Sectional	114 (8–10 years old)	Parents/tutors of participants signed informed consent	Male: 9.53 years (SD, 0.64) Female: 9.54 years (SD, 0.6)	Male: 18.8 (SD, 4.52) Female:18.4 (SD, 4.19)	PAQ-A: Male: 3.82(0.76) Female:3.56 (0.83)	NR	KIDMED index	16 dichotomous items (yes/no): 12 items: behaviors consistent with the Mediterranean diet 4 items: behaviors inconsistent with the Mediterranean diet	KIDSCREEN-27	5 Dimensions: 1. Physical well-being 2. Psychological well-being 3. Autonomy and relationship with parents 4. Social relationships and Peer pressure 5. School environment	NR	NR
Caamaño-Navarrete et al., 2021 [22]	Chile	Cross-Sectional	634 (11–13 years old)	Chilean schoolchildren aged between 11 and 13, without musculoskeletal disorders or any other known medical conditions that may alter the participants’ health and PA levels	55.5% boys Mean age: 11.95 years (SD, 0.85)	21.6 (SD, 4.58)	2.64 h/week (1.42)	NR	KIDMED index**	Each item has a score of + 1 or – 1, depending on whether it approximates the ideal of the MD. The sum of all values from the administered test is categorized into three different levels: (1) > 8, optimal MD; (2) 4–7, improvement needed to adjust intake to Mediterranean patterns; and (3) ≤ 3, very low diet quality	KIDSCREEN-10	It contains 10 items regarding family life, peers, and school life	NR	NR
Mitri et al., 2021 [23]	Lebanon	Cross-Sectional	798 (11–18 years old)	Students suffering from physical disabilities or who were absent on the days of the data collection were not invited to participate in the study	52.9% boys Mean age: 15 years (SD, 2.07)	NR	PAQ C/A: 2.46 (0.75)	Normal weight: 55.4% Overweight: 20.2% Obesity: 19.3%	KIDMED index	16 dichotomous items (yes/no): 12 items: behaviors consistent with the Mediterranean diet 4 items: behaviors inconsistent with the Mediterranean diet	KIDSCREEN-27	5 Dimensions: 1. Physical well-being 2. Psychological well-being 3. Autonomy and relationship with parents 4. Social relationships and Peer pressure 5. School environment	School type, grade, age, skipping meals, father's education level, number of meals, number of meals with family, and physical activity	Self-funded
Mozzillo et al., 2021 [24]	Italy	Cross-sectional	420 (13–17 years old)	Age 13.0–17.0 years; Caucasian ethnicity; overweight or obesity; first visit at the outpatient clinic. The exclusion criteria were secondary causes of obesity (genetic, endocrine, or iatrogenic forms) and other chronic diseases or mental illnesses	44.5% boys Median age: 14.0 years (IQR: 13.2; 15.0)	31.2 (IQR: 28.7; 34.2)	0 h/week (0–3)	82.3 (IQR: 73.6; 93.2)	KIDMED index	16 dichotomous items (yes/no): 12 items: behaviors consistent with the Mediterranean diet 4 items: behaviors inconsistent with the Mediterranean diet	Pediatric Quality of Life Inventory (PedsQL)	4 Dimensions: 1. Physical functioning 2. Emotional functioning 3. Social functioning 4. School functioning	BMI, father’s education level (year), mother’s education level (year), exercise (hours/week)	Self-funded

Data are presented as mean (SD) or median (IQR)

^*^Originally a longitudinal study, it was considered the baseline cross-sectional cohort

^**^The original article states that the Kreece Plus scale was used, but the KIDMED scale is cited and used

^†^Obesity classification (%) IOTF cutoff points, except in Delgado et al. and Mitri et al. classified with CDC criteria

^‡^ Self-reported score

*BM*I (body mass index), *MD* (Mediterranean diet), *HRQoL* (Health-Related Quality of Life), *KIDMED* (Mediterranean Diet Quality Index), *KIDSCREEN* (KIDSCREEN test), *PedsQL* (Pediatric Quality of Life Inventory), *Krece Plus* (Krece PLus test), *SD* (standard deviation), *IQR* (interquartile range), *NR* (not reported), *PAQ-C* (Physical Activity Questionnaire for Older Children, a five-point scale), *PAQ-A* (Physical Activity Questionnaire for Adolescents, a five-point scale), *PAQ C/A* (Physical Activity Questionnaires for Children and Adolescents, a five-point scale)

### Risk of bias in studies

Six studies reported their methodologies for representativeness of the sample and sample size calculation, but only three reported the nonresponse rate of the participants included in the study. All the studies collected data by self-reporting and reported comparability between both groups, and three took into account the confounding factors. On the other hand, six studies report a misdescribed or incomplete statistical analysis (lack of confidence intervals or p-values for measures of association). In this way, four studies had a low risk of bias, while seven had a high risk of bias (Table 2).

**Table 2:** Risk of bias of the included studies

Author, publication year	Study design	Selection				Comparability	Outcome		Score	Interpretation
		**Representativeness of the sample**	**Sample size**	**Nonrespondents**	**Ascertainment of exposure**	**Based on design and analysis**	**Assessment of outcome**	**Statistical test**		
Costarelli et al., 2012 [14]	Cross-sectional		+		+ +	+	+		5	High risk of bias
Muros et al., 2016 [15]	Cross-sectional	+	+		+ +	+ +	+		7	**Low risk of bias**
Zervaki et al., 2017 [16]	Cross-sectional				+ +	+	+		4	High risk of bias
Evaristo et al., 2018 [17]	Cross-sectional	+	+	+	+ +	+ +	+	+	9	**Low risk of bias**
Esteban-Gonzalo et al., 2019* [18]	Cross-sectional				+ +	+	+	+	5	High risk of bias
Ferrer-Cascales et al., 2019 [19]	Cross-sectional	+	+	+	+ +	+ +	+		8	**Low risk of bias**
Delgado-Floody et al., 2020 [20]	Cross-sectional	+			+ +	+	+	+	6	High risk of bias
Rodriguez-Rosado et al., 2020 [21]	Cross-sectional				+ +	+	+		4	High risk of bias
Caamaño-Navarrete et al., 2021 [22]	Cross-sectional		+	+	+ +	+	+	+	7	**Low risk of bias**
Mitri et al., 2021 [23]	Cross-sectional	+	+		+ +	+	+		6	High risk of bias
Mozzillo et al., 2021 [24]	Cross-sectional	+			+ +	+	+	+	6	High risk of bias

^*^Originally a longitudinal study, it was considered the baseline cross-sectional cohort

### Results of individual studies

Ten studies evaluated MD adherence with the KIDMED index, while the remaining one used the Krece Plus test. Regarding HRQoL, four studies used the score KIDSCREEN-10, five the KIDSCREEN-27, one the KIDSCREEN-52, and one the PedsQL (Table 3). The mean MD scores ranged from 5.1 to 7.87, while the mean HRQoL scores were 37.37 (KIDSCREEN-10) and between 39.1 and 42.0 (KIDSCREEN-52), with a median of 75 (PedsQL). Ten studies analyzed the association between MD adherence and HRQoL with linear regression reporting β-values with 95% confidence intervals (CIs); the remaining study evaluated the association using logistic regression, reporting the odds ratio (OR). Eight studies used adjusted models for sex, BMI, overweight or obesity, physical activity, number of meals, pubertal stage, father’s or mother’s education level, socioeconomic status, sleep duration, school type, or grade. Of the eight studies that reported the value of the correlation with the HRQoL general score, four [14–17] showed positive associations of MD adherence with higher levels of HRQoL with statistically significant values; the β-value ranged from 0.13 to 0.26. In contrast, the study that evaluated the OR [24] reported a value of 0.878 (95% CI, 0.804–0.959) between better MD adherence and high/intermediate HRQoL (for each increase in one unit of the KIDMED score, the odds of "low total functioning" will decrease by 12%). Of the other three studies with no statistically significant results, two found a positive association, and one found a negative association between MD and HRQoL.

**Table 3:** Main findings of the included studies

Study	MD adherence score	HRQoL score	HRQoL dimensions score	Correlation	
				**β (95% CI)**	***p* value**
**Krece Plus vs. KIDSCREEN-10**					
Delgado-Floody et al., 2020 [20]	NR	NR	NR	For general score: NR For score by ethnicity Ethnic ascendant: 0.12 (0.04; 0.25) Nonethnic ascendant: 0.01 (-0.07; 0.09)	Ethnic ascendant: 0.063 Nonethnic ascendant: 0.763
**KIDMED vs. KIDSCREEN-27**					
Costarelli et al., 2012 [14]	6.3 (SD, 2.4)	50.1 (SD, 7.5)	1. Physical well-being: 50.3 (SD, 9.8) 2. Psychological well-being: 50 (SD, 10.0) 3. Autonomy and relationship with parents: 50.05 (SD, 10.01) 4. Social relationships and peer pressure: 50 (SD, 9.8) 5. Peers and school environment: 50.3 (SD, 9.8)	For general score: 0.21	< 0.001
Muros et al., 2016 [15]	7.87 (SD, 2.08)	52.96 (SD, 8.21)	NR	For general score: 0.142	< 0.01
Zervaki et al., 2017 [16]	5.1 (SD, 1.8)	50 (SD, 10)	1. Physical well-being: 50.3 (SD, 9.9) 2. Psychological well-being: 49.9 (SD, 10.1) 3. Autonomy and relationship with parents: 50 (SD, 10) 4. Social relationships and peer pressure: 50.1 (SD, 9.8) 5. Peers and school environment: 50 (SD, 9.9)	For general score: 0.13	0.049
Rodriguez-Rosado et al., 2020 [21]	Male: 6.20 (SD, 2.43) Female: 6.32 (SD, 2.44)	Male: 82.1 (SD, 11.6) Female: 82.42 (SD, 10.88)	Male: 1. Physical well-being: 85.3 (SD, 14.3) 2. Psychological well-being: 83.8 (SD, 11.7) 3. Autonomy and relationship with parents: 75.6 (SD, 18.1) 4. Social relationships and peer pressure: 85.8 (SD, 19.4) 5. Peers and school environment: 84.1 (SD, 13.2) Female: 1. Physical well-being: 83.5 (SD, 15.1) 2. Psychological well-being: 83.2 (SD, 10.6) 3. Autonomy and relationship with parents: 72.4 (SD, 15.4) 4. Social relationships and peer pressure: 88.1 (SD, 13.6) 5. Peers and school environment: 88.8 (SD, 13.6)	For general score: 0.125 For score by dimensions 1. General health: NR 2. Physical well-being: 0.179 3. Psychological well-being: 0.121 4. Autonomy and relationship with parents: 0.49 5. Social relationships and peer pressure: 0.98 6. Peers and school environment: 0.209	General score: 0.130 1. General health: NR 2. Physical well-being: 0.035 3. Psychological well-being: 0.173 4. Autonomy and relationship with parents: 0.595 5. Social relationships and peer pressure: 0.264 6. Peers and school environment: 0.012
Caamaño-Navarrete et al., 2021 [22]	6.11 (SD, 2.34)	37.37 (SD, 4.77)	NR	For general score: 0.11 (0.00; 0.22)	0.059
Mitri et al., 2021 [23]	Low (≤ 3): 233 (29.2%) Moderate (4–7): 459 (57.5%) Optimal (≥ 8): 106 (13.3%)	45.81 (SD, 6)	1. Physical well-being: 44.85 (SD, 8.44) 2. Psychological well-being: 37.38 (SD, 4.41) 3. Autonomy and relationship with parents: 49.12 (SD, 9.58) 4. Social relationships and peer pressure: 48.91 (SD, 11.51) 5. Peers and school environment: 48.72 (SD, 11.56)	For general score: -0.069	0.093
**KIDMED vs. KIDSCREEN-10**					
Evaristo et al., 2018 [17]	7.1 (SD, 2.1)	39.1 (SD, 5.6)	NR	For general score: 0.259 (0.096; 0.421)	0.002
Esteban-Gonzalo et al., 2019* [18]	Primary school: Male: 5.9 (SD, 2.4) Female: 6.5 (SD, 2.2) Secondary school: Male: 6.6 (SD, 2.3) Female: 6.4 (SD, 2.2)	Primary school: Male: 41.5 (SD, 4.5) Female: 42.0 (SD, 5.0) Secondary school: Male: 39.6 (SD, 5.2) Female: 38.1 (SD, 5.3)	NR	Primary school: Male: 0.11 (-0.13; 0.36) Female: 0.08 (-0.19; 0.37) Secondary school: Male: 0.46 (0.25; 0.66) Female: 0.41 (0.21; 0.61)	Primary school: Male: 0.361 Female: 0.547 Secondary school: Male: < 0.001 Female: < 0.001
**KIDMED vs. KIDSCREEN-52**					
Ferrer-Cascales et al., 2019 [19]	NR	NR	1. Physical well-being: 18.05 (SD, 3.9) 2. Psychological well-being: 23.33 (SD, 4.8) 3. Mood and emotions: 26.79 (SD, 5.8) 4. Self-perception: 19.49 (SD, 3.5) 5. Autonomy: 19.01 (SD, 4.24) 6. Parent relations and home life: 24.66 (SD, 4.88) 7. Financial resources: 11.9 (SD, 2.86) 8. Social support and peers: 25.05 (SD, 4.05) 9. School environment: 21.39 (SD, 4.53) 10. Social acceptance: 13.19 (SD, 2.23)	For general score: NR For score by dimensions 1. Physical well-being: 0.812 2. Psychological well-being: 1.28 3. Mood and emotions: 1.62 4. Self-perception: 0.603 5. Autonomy: 1.06 6. Parent relations and home life: 1.23 7. Financial resources: 0.433 8. Social support and peers: 0.756 9. School environment: 0.934 10. Social acceptance: 0.212	1. Physical well-being: 0.00001 2. Psychological well-being: 0.00001 3. Mood and emotions: 0.00001 4. Self-perception: 0.00001 5. Autonomy: 0.00001 6. Parent relations and home life: 0.00001 7. Financial resources: 0.00001 8. Social support and peers: 0.00001 9. School environment: 0.00001 10. Social acceptance: 0.00001
**KIDMED vs. PedsQL**					
Mozzillo et al., 2021 [24]	5 (IQR:3; 7)	75 (IQR: 65; 83)	1. Physical functioning: 75 (IQR: 66; 84) 2. Emotional functioning: 70 (IQR: 55; 80) 3. Social functioning: 85 (IQR: 70; 95) 4. School functioning: 70 (IQR: 55; 85)	For general score Odds ratio: 0.878 (0.804; 0.959)	0.004

Data are presented as mean (SD) or median (IQR)

^*^Originally a longitudinal study, it was considered the baseline cross-sectional cohort

*MD* (Mediterranean diet), *HRQoL* (Health-Related Quality of Life), *NR* (no report), CI (confidence interval)

Two studies reported the association for each dimension of HRQoL; one finding a statistically significant association with all dimensions (physical well-being, psychological well-being, mood and emotions, self-perception, autonomy, parent relations and home life, financial resources, social support and peers, school environment, and social acceptance), and the other found association only with two (physical well-being, and peers and school environment). Finally, two studies reported the association between ethnic ascendant and children's primary or secondary school; a statistically significant association was found only in secondary school children in the latter study (male, β = 0.46; 95% CI, 0.25–0.66; female, β = 0.41; 95% CI, 0.21–0.61).

## Discussion

### Summary of the results

We included 11 studies (10 cross-sectional analytical studies and one cohort) that evaluated the association between adherence to MD and HRQoL in children and adolescents. Most of the studies were conducted in Europe. Of the included studies, only four demonstrated a low risk of bias. A statistically significant positive association between adherence to MD and HRQoL was observed in five of the included studies [14–17, 24], reporting that better adherence to MD improves HRQoL levels. In addition, one study demonstrated this association in all dimensions of HRQoL [19] and another only in adolescents [18].

### Comparison with other studies

A previous systematic review evaluating the influence of diet quality and dietary behavior on HRQoL in children and adolescents [25] identified only three of our included studies [14–16], where they highlight that adolescents with better adherence to the MD experienced better HRQoL than those with low adherence. This finding is consistent with our results, where five of the included studies [14–17, 24] showed that adherence to MD was positively associated with HRQoL, with β values ranging from 0.13 to 0.26 points. Furthermore, in a systematic review [4] that included 43,445 adults, it was observed that there is an association between a higher HRQoL score and greater adherence to a MD pattern than those in people with a Western dietary pattern, who scored lower in HRQoL. However, it is necessary to determine whether a MD could improve the QoL of the child, or on the contrary, adherence to the MD is an expression of the child’s QoL. A recent study evaluated the association of parental socioeconomic status with the child’s adherence to the MD, observing that parents with lower socioeconomic status had children who ate a higher proportion of sweets and junk food, and less proportion of fruits and vegetables [26].

MD adherence may affect QoL through different pathways. It can potentiate overall children's health while also ameliorating some pediatric disorders [27]. Higher MD adherence may lead to better physical activity, sleep quality, and satisfaction with body image [28–30]. Higher MD adherence is also correlated with less frequency of functional constipation [31]. Functional constipation is correlated with a poorer QoL [32]. We might think that adherence to the MD mediates these effects through better body weight maintenance; however, a recent longitudinal study in Norway shows that the overall quality of the diet, rather than adherence to a specific dietary pattern, is associated with a lower risk of obesity [33].

### Implications for clinical practice

In specific populations such as children and adolescents, the current data come mainly from observational studies as in our review; therefore, RCTs are needed. Despite this, current evidence suggests that the MD would have a beneficial effect during child developmental stages leading to a better QoL [34].

Improving eating habits toward a MD pattern may lead to greater physical activity in children and adolescents, leading to a decrease in sedentary behaviors and better overall health [35]. Moreover, although adherence to the MD is associated with higher costs in children's diets [36], according to cost–benefit analyses, it is cost-effective in the long term because it prevents future degenerative pathologies [35]. Thus, understanding the behaviors associated with adherence to MD in the pediatric population could be essential for the appropriate and specific design of public health interventions, contributing to the early adoption of healthy habits to reduce the negative impact of Western dietary patterns [34].

### Limitations and strengths

The heterogeneity of the instruments used when evaluating HRQoL, inclusion criteria, insufficient data, and outcome measures of some studies that were included prevented a quantitative synthesis of the data and the performance of a meta-analysis. Four of the 11 studies have a low risk of bias. Thus, this could be a concern. However, we are confident that there is a true association, given that three of these four studies highlighted a direct relationship. In addition, we only identified observational studies mostly in the European population; therefore, our conclusions could not be fully extrapolated to other regions.

However, this review has strengths that are worth mentioning. We conducted a comprehensive search strategy in several databases without language restriction. This exhaustive literature search had a rigorous selection process for identifying eligible studies using predefined inclusion and exclusion criteria. We also searched the articles cited in the included studies, which allowed us to find the articles contained in previous systematic reviews [37, 38], and others [25].

Finally, this research gives way to future work that will strengthen the evidence regarding the comparison of QoL with dietary patterns and preferences. Since there is a positive effect of healthy diets on HRQoL, several studies relate QoL to domains such as physical activity, school and emotional development, as well as psychosocial life [39, 40].

## Conclusion

The findings of our systematic review suggest the positive correlation between adherence to MD and HRQoL in children and adolescents. Future research is needed to strengthen the evidence of the relationship between QoL and other dietary patterns, as well as the adaptation of the MD in different regions of the world.

## Supplementary Information

**Additional file 1:** Search strategy

**Additional file 2:** Reasons for the exclusion of studies from the systematic review

## Data Availability

All data generated or analyzed during this study are included in this published article.

## Abbreviations

QoL: Quality of life
HRQoL: Health-related quality of life
MD: Mediterranean diet
NOS: Newcastle–Ottawa Scale
