# Supplementary material for: Adherence to Mediterranean diet associated with health-related quality of life in children and adolescents: a systematic review
Source: BMC Nutr. 2022 Jun 23;8:57. doi: 10.1186/s40795-022-00549-0 (PMC9219125; doi:10.1186/s40795-022-00549-0)
Supplement: Supplementary file 1 — Additional file 1: Search strategy [file 40795_2022_549_MOESM1_ESM.pdf]

Additional file 1. Search strategy

Date: February 10, 2021
Update: September 11, 2021
Filters: None

Database	Search strategy	Results
PubMed	#1: Diet, Mediterranean[MH] OR Mediterranean Diet*[TIAB] OR Mediterranean Diet*[OT]<br>#2: Quality of Life[MH] OR Quality of Life[TIAB] OR Life Quality[TIAB] OR HRQOL[TIAB] OR Quality of Life[OT] OR Life Quality[OT] OR HRQOL[OT]<br>#3: Child[MH] OR Child[TIAB] OR Children[TIAB] OR Child[OT] OR Children[OT] OR Pediatrics[MH] OR Pediatric*[TIAB] OR Paediatric*[TIAB] OR Pediatric*[OT] OR Paediatric*[OT] OR Kids[TIAB] OR Kids[OT]<br>#4: Adolescent[MH] OR Adolescen*[TIAB] OR Teen*[TIAB] OR Youth*[TIAB] OR Adolescen*[OT] OR Teen*[OT] OR Youth*[OT]<br>#5: #1 AND #2 AND (#3 OR #4)	46 results
Scopus	#1: TITLE-ABS-KEY ("mediterranean diet*" OR meddiet)<br>#2: TITLE-ABS-KEY("Quality of Life" OR "Life Quality" OR HRQOL)<br>#3: TITLE-ABS-KEY((Child* AND NOT (Childbirth OR Childbearing)) OR Pediatric* OR Paediatric* OR Kid OR Kids)<br>#4: TITLE-ABS-KEY(Adolescen* OR Teen* OR Youth*)<br>#5: #1 AND #2 AND (#3 OR #4)	68 results
Web of Science	#1: TS=(“Quality of Life” OR “Life Quality” OR “HRQOL”)<br>#2: TS=("mediterranean diet*" OR meddiet)<br>#3: TS=((Child* NOT (Childbirth OR Childbearing)) OR Pediatric* OR Paediatric* OR Kid OR Kids)<br>#4: TS=(Adolescen* OR Teen* OR Youth*)<br>#5: #1 AND #2 AND (#3 OR #4)	95 results
Embase	#1: 'quality of life':ti,ab,kw OR 'life quality':ti,ab,kw OR 'hrqol':ti,ab,kw<br>#2: 'mediterranean diet*':ti,ab,kw OR meddiet:ti,ab,kw<br>#3: child*:ti,ab,kw NOT (childbirth:ti,ab,kw OR childbearing:ti,ab,kw)<br>#4: pediatric*:ti,ab,kw OR paediatric*:ti,ab,kw OR kid:ti,ab,kw OR kids:ti,ab,kw<br>#5: adolescen*:ti,ab,kw OR teen*:ti,ab,kw OR youth*:ti,ab,kw<br>#6: #1 AND #2 AND (#3 OR #4 OR #5)	25 results
Cochrane Library	#1: MeSH descriptor: [Diet, Mediterranean] explode all trees<br>#2: MeSH descriptor: [Quality of Life] explode all trees<br>#3: MeSH descriptor: [Child] explode all trees<br>#4: MeSH descriptor: [Pediatrics] explode all trees<br>#5: MeSH descriptor: [Adolescent] explode all trees<br>#6: "Quality of life":ti,ab OR Life Quality:ti,ab OR HRQOL:ti,ab<br>#7: Mediterranean Diet*:ti,ab<br>#8: Child:ti,ab OR Children:ti,ab OR Pediatric*:ti,ab OR Paediatric*:ti,ab OR Kids:ti,ab OR kid:ti,ab<br>#9: Adolescen*:ti,ab OR Teen*:ti,ab OR Youth:ti,ab<br>#10: #1 OR #7<br>#11: #2 OR #6<br>#12: #3 OR #4 OR #5 OR #8 OR #9<br>#13: #10 AND #11 AND #12	20 results
Ovid MEDLINE	#1: ((Mediterranean Diet or Mediterranean Diets) and (Quality of Life or Life Quality or HRQOL) and (Child or Children or Pediatric* or Paediatric* or Kid or Kids or Adolescen* or Teen* or Youth*)).ab.	31 results
