# Supplementary material for: Adherence to Mediterranean diet associated with health-related quality of life in children and adolescents: a systematic review
Source: BMC Nutr. 2022 Jun 23;8:57. doi: 10.1186/s40795-022-00549-0 (PMC9219125; doi:10.1186/s40795-022-00549-0)
Supplement: Supplementary file 2 — Additional file 2: Reasons for the exclusion of studies from the systematic review [file 40795_2022_549_MOESM2_ESM.pdf]

Additional File 2: Reasons for the exclusion of studies from the systematic review

Author	Year	Title	Reason for exclusion
Morales-Suárez et al.	2015	Comparative study of lifestyle: eating habits, sedentary lifestyle and anthropometric development in Spanish 5-to 15-yr-olds	The correlation between the Mediterranean diet and HRQoL not analyzed
Knox et al. (a)	2017	Gender and school-stage associations with health-related behaviours and health-related quality of life in Spanish children	The correlation between the Mediterranean diet and HRQoL not analyzed
Knox et al. (b)	2017	Association of lifestyle behaviours with self-esteem through health-related quality of life in Spanish adolescents	Statistical inconsistencies in their results
Evaristo et al.	2018	Associations between physical fitness and adherence to the Mediterranean diet with health-related quality of life in adolescents: results from the LabMed Physical Activity Study	The correlation between the Mediterranean diet and HRQoL not analyzed
Delgado-Floody et al.	2018	The social and psychological health of children is associated with Mediterranean diet adherence items, cardiorespiratory fitness, and lifestyle	Data pertaining to the same population of a study already included
González-Valero et al.	2018	Eating habits and lifestyles in schoolchildren from Granada (Spain). A pilot study	Mediterranean diet or HRQoL not evaluated
Zurita-Ortega et al.	2018	Physical activity and health-related quality of life in schoolchildren: structural equations analysis	Statistical inconsistencies in their results
Agakidis et al.	2019	Mediterranean diet adherence is associated with lower prevalence of functional gastrointestinal disorders in children and adolescents	Mediterranean diet or HRQoL not evaluated
Delgado-Floody et al.	2019	The association between children's food habits, anthropometric parameters and health-related quality of life in Chilean school-age children	Statistical inconsistencies in their results
Galán-López et al.	2020	Adherence to the Mediterranean diet, motives for physical exercise and body composition in Icelandic adolescents: The AdolesHealth Study	Mediterranean diet or HRQoL not evaluated
Jiménez-Boraita et al. (a)	2020	Health-related quality of life and lifestyle habits: differences between migrant and native adolescents	The correlation between the Mediterranean diet and HRQoL not analyzed
Jiménez-Boraita et al. (b)	2020	Gender differences relating to lifestyle habits and health-related quality of life of adolescents	Data pertaining to the same population of a study already included
Jiménez-Boraita et al. (c)	2020	Factors associated with adherence to a Mediterranean diet in adolescents from the northern region of Spain	HRQoL presented a value that was not plausible
Silva et al.	2020	Health-related quality of life of Portuguese children and adolescents according to diet quality and food intake	Mediterranean diet or HRQoL not evaluated
Solera-Sanchez et al.	2020	Health-related quality of life in adolescents: individual and combined impact of health-related behaviors (DADOS study)	Statistical inconsistencies in their results
Jiménez-Boraita et al.	2021	Quality of life related to health and habits: differences between adolescents in rural and urban environments	The correlation between the Mediterranean diet and HRQoL not analyzed
Mastorci et. al.	2021	Health-related quality of life in Italian adolescents during Covid-19 outbreak	The correlation between the Mediterranean diet and HRQoL not analyzed
Dragun et. al.	2021	Have Lifestyle Habits and Psychological Well-Being. Changed among Adolescents and Medical Students Due to COVID-19 Lockdown in Croatia?	The correlation between the Mediterranean diet and HRQoL not analyzed

HRQoL (Health-Related Quality of Life)
